# Intestinal GATA4 deficiency protects from diet-induced hepatic steatosis

**DOI:** 10.1016/j.jhep.2012.06.028

**Published:** 2012-11

**Authors:** Jay V. Patankar, Sascha Obrowsky, Prakash Doddapattar, Gerald Hoefler, Michele Battle, Sanja Levak-Frank, Dagmar Kratky

**Affiliations:** 1 Institute of Molecular Biology and Biochemistry, Medical University of Graz, Austria; 2 Institute of Pathology, Medical University of Graz, Austria; 3 Department of Cell Biology, Neurobiology and Anatomy, Medical College of Wisconsin, USA

**Keywords:** DNL, *de novo* lipogenesis, TG, triglycerides, NAFLD, non-alcoholic fatty liver disease, WTD, Western-type diet, ACC, acetyl-CoA carboxylase alpha, MCDD, methionine and choline-deficient diet, GLP-1, glucagon-like peptide-1, IIS, ileal interposition surgery, *GATA4*iKO, intestine-specific *GATA4* deficiency, ALT, alanine aminotransferase, AST, aspartate transaminase, LDH, lactate dehydrogenase, FFA, free fatty acids, TBARS, thiobarbituric acid reactive substances, AMPK, AMP-activated protein kinase, p, phosphorylated, p38, p38 mitogen-activated protein kinase, Pparg, peroxisome proliferator-activated receptor gamma, α-SMA, alpha-smooth muscle actin, SREBP-1c, sterol regulatory element-binding protein-1c, HSC, hepatic stellate cells, *GATA4*, Non-alcoholic fatty liver disease, GLP-1, Ileal interposition surgery, *De novo* lipogenesis

## Abstract

**Background & Aims:**

GATA4, a zinc finger domain transcription factor, is critical for jejunal identity. Mice with an intestine-specific GATA4 deficiency (*GATA4*iKO) are resistant to diet-induced obesity and insulin resistance. Although they have decreased intestinal lipid absorption, hepatic *de novo* lipogenesis is inhibited. Here, we investigated dietary lipid-dependent and independent effects on the development of steatosis and fibrosis in *GATA4*iKO mice.

**Methods:**

*GATA4*iKO and control mice were fed a Western-type diet (WTD) or a methionine and choline-deficient diet (MCDD) for 20 and 3 weeks, respectively. Functional effects of *GATA4*iKO on diet-induced liver steatosis were investigated.

**Results:**

WTD-but not MCDD-fed *GATA4*iKO mice showed lower hepatic concentrations of triglycerides, free fatty acids, and thiobarbituric acid reactive species and had reduced expression of lipogenic as well as fibrotic genes compared with controls. Reduced nuclear sterol regulatory element-binding protein-1c protein levels were accompanied by lower lipogenic gene expression. Oil red O and Sirius Red staining of liver sections confirmed the observed reduction in hepatic lipid accumulation and fibrosis. Immunohistochemical staining revealed an increased number of jejunal glucagon-like peptide 1 (GLP-1) positive cells in *GATA4*iKO mice. Consequently, we found enhanced phosphorylation of hepatic AMP-activated protein kinase and acetyl-CoA carboxylase alpha.

**Conclusions:**

Our results provide strong indications for a protective effect of intestinal GATA4 deficiency on the development of hepatic steatosis and fibrosis via GLP-1, thereby blocking hepatic *de novo* lipogenesis.

## Introduction

Metabolic syndrome is a state of metabolic deregulation characterized by obesity, hyperlipidemia, atherosclerosis, glucose intolerance, and hepatic steatosis [1]. A key mechanism contributing to the development of metabolic syndrome is the rate of hepatic *de novo* lipogenesis (DNL) [2]. Hepatic DNL contributes only 5% of liver triglycerides (TG) under healthy circumstances but up to 30% in case of non-alcoholic fatty liver disease (NAFLD) [3]. The derangement of DNL in murine models of high fat diet-induced hepatic steatosis underscores the intestinal contribution in regulating hepatic lipid metabolism. Western-type diet (WTD), which is generally used to induce atherosclerosis, insulin resistance, and metabolic syndrome in mice, also causes non-alcoholic steatohepatitis [4,5]. WTD contains 43% of calories in the form of carbohydrates and dietary carbohydrates are known to activate hepatic DNL via acetyl-CoA carboxylase (ACC) [6]. Methionine and choline-deficient diet (MCDD) is widely used as a model of hepatic steatosis and fibrosis by inhibiting the release of very low density lipoproteins and by decreasing mitochondrial fatty acid oxidation [7].

Ileum of the small intestine is an important endocrine organ, which signals the dietary status to other organs including the liver by the release of hormones such as glucagon-like peptide-1 (GLP-1) [8,9]. A recent meta-analysis of 15 studies has investigated the effect of various bariatric procedures on NAFLD. The study has concluded that bariatric surgery ameliorates steatosis in 92%, steatohepatitis in 81% and leads to complete resolution in 70% of patients [10]. Ileal interposition surgery (IIS) is one of the bariatric procedures that mitigate the metabolic syndrome [11]. IIS in rats improves glucose tolerance and increases synthesis and release of GLP-1 and peptide YY [12,13]. Kohli *et al.* have recently reported that the cycling of bile acids is increased in rats that have undergone IIS and that these mice are protected from obesity-associated co-morbidities [14]. These studies provide evidence that postsurgical changes in intestinal anatomy and function, especially earlier exposure of the ileum to nutrients together with alterations in the secretion of enteric hormones, contribute to improve glucose tolerance and lipid homeostasis after IIS. However, the long-term effects this surgical procedure may exert on hepatic lipid metabolism, steatosis, fibrosis, and inflammation are still unknown.

GATA4, a zinc finger domain transcription factor expressed throughout the small intestine except the distal ileum, plays an important role in maintaining jejuno-ileal differences in absorptive enterocyte gene expression [15,16]. Using a Villin-Cre approach, Battle *et al.* generated intestine-specific *GATA4* knockout (*GATA4*iKO) mice and showed that 47% of ileal genes were ectopically expressed in the jejunum of these mice [15]. This jejuno-ileal transition resembles IIS. We have previously demonstrated that *GATA4*iKO mice were resistant to diet-induced obesity and insulin resistance owing to reduced intestinal lipid absorption and increased GLP-1 release [17].

In the present study, we have investigated the effect of intestinal GATA4 deficiency on hepatic steatosis and fibrosis. Using two dietary conditions, we provide evidence that decreased hepatic DNL leads to protection from diet-induced NAFLD in *GATA4*iKO mice.

## Materials and methods

### Animals and diets

Generation of *Gata4^loxP/null^Villin-Cre* knockout and *Gata4^loxP/+^ Villin-Cre* control mice have previously been described [15]. All experiments were performed using male mice. Mice had free access to food and water under a 12 h light/12 h dark cycle in a temperature-controlled environment. Individually housed knockout and control littermates were fed a normal chow diet (11.9% caloric intake from fat, Ssniff®, Soest, Germany) or switched to WTD (TD88137 mod.; Ssniff®, Soest, Germany) for 20 or MCDD (A02082002B, Research Diets, Inc. New Brunswick, NJ) for 3 weeks. WTD contained 21% (weight/weight) crude fat and 0.15% (weight/weight) cholesterol with ≈ 4.53 kcal/g (42% of calories from crude fat, 15% from protein, and 43% from carbohydrate). Features of metabolic syndrome along with steatohepatitis are reported to develop after long-term WTD feeding [4]. MCDD is reported to induce severe steatohepatitis in a relatively shorter time period from 3 to 6 weeks [7]. Appropriate feeding regimens were chosen accordingly. Animal experiments were performed in accordance with the standards established by the Austrian Federal Ministry of Science and Research, Division of Genetic Engineering and Animal Experiments (Vienna, Austria).

### Biochemical analyses

Blood was drawn via retro-orbital puncture and plasma was isolated for analysis. Levels of alanine aminotransferase (ALT), aspartate transaminase (AST), and lactate dehydrogenase (LDH) were routinely measured in 4 mice per genotype.

### Oral fat tolerance test

Blood was drawn via retro-orbital puncture before and after an oral gavage of 200 μl of corn oil at pre-established time points and plasma was isolated. Plasma TG (n = 8/group) was estimated according to manufacturer’s protocol (DiaSys, Holzheim, Germany).

### Hepatic TG and free fatty acid (FFA) estimation

Hepatic TG and FFA concentrations were determined from Folch extracts using enzymatic kits according to manufacturer’s protocols (DiaSys, Holzheim, Germany; Wako Chemicals GmbH, Neuss, Germany). Briefly, liver samples from *GATA4*iKO and control mice fed WTD (n = 5/group), MCDD (n = 4/group) or chow diet (n = 5/group) were weighed and homogenized, and lipids were extracted in chloroform:methanol (2:1) in a volume 20 times the weight of the sample. Lipid extracts were then solubilized in freshly prepared 0.2% Triton X-100 in chloroform, dried under a gentle stream of nitrogen, and resuspended in 200 μl sterile phosphate-buffered saline.

### Assay for hepatic lipid peroxidation levels

Hepatic levels of thiobarbituric acid reactive substances (TBARS) were determined using the Cayman TBARS Assay kit (Cayman Chemicals, Ann Arbor, MI). Briefly, 25 mg of liver tissue from WTD-fed control and *GATA4*iKO mice (n = 5/group) was homogenized in 250 μl of radio-immunoprecipitation assay buffer supplemented with protease inhibitors (Roche, Indianapolis, IN) and sonicated on ice for 15 s at medium power. Supernatants were separated by centrifugation at 3500 rpm for 10 min at 4 °C. One hundred μl of each sample and standard was mixed with 4 ml color reagent containing 100 μl of sodium dodecyl sulfate solution. Samples were boiled for 1 h, and the reaction was stopped by placing samples on ice for 10 min. Thereafter, samples were centrifuged at 3500 rpm for 10 min at 4 °C. The absorbance was measured at 530 nm. Results were expressed as μM of malondialdehyde-adduct per g liver weight.

### Assay for hepatic 4-hydroxyproline levels

Hepatic levels of 4-hydroxyproline were measured as previously described [18]. Briefly, liver tissues were homogenized in 2 N NaOH and autoclaved at 120 °C for 25 min for alkaline hydrolysis. The hydrolysates were cooled to RT and filtered through Whatmann filter paper No. 1 to remove particulate matter. Standard dilutions of 4-hydroxyproline (Sigma Aldrich, GmbH, Germany), samples and blanks in acetate-citrate buffer pH 6.5 were mixed in freshly prepared chloramine T and allowed to react at room temperature for 25 min. Color development was achieved by the addition of fresh Ehrlich’s aldehyde reagent and incubation at 65 °C for 20 min. Absorbances were measured at 550 nm. Results were plotted as mg collagen/g liver using the following formula:

g collagen=(g 4-hydroxyproline/ml)∗dilution factor∗conversion factor(7.5)

### Histochemistry and immunohistochemistry

For conventional light microscopy, livers of WTD-fed control and *GATA4*iKO mice (n = 3/group) and MCDD-fed control and *GATA4*iKO mice (n = 4/group) were fixed in 4% neutral buffered formaldehyde solution for 24 h and embedded in paraffin. Five μm sections were deparaffinized and subjected to routine haematoxylin and eosin, periodic acid Schiff and Sirius Red staining. For histochemical staining with Oil Red O, 7 μm cryosections were prepared and stained according to protocol; nuclei were counter-staining with Mayer’s haematoxylin. For the detection of GLP-1 positive cells in the jejunum of control and *GATA4*iKO mice, harvested jejunum was longitudinally exposed and luminal contents were washed in PBS. The tissue was immediately fixed in 10% formaldehyde for 24 h and embedded in paraffin. Five μm sections were processed for staining. Sections were deparaffinized and blocked in 2% pre-immune goat serum. Sections were incubated with anti-GLP-1 antibody (1:500; Phoenix Pharmaceuticals Inc., Burlingame, CA) overnight at 4 °C, washed, incubated with biotinylated goat anti-rabbit secondary antibody followed by signal enhancement using the ABC reagent (Vector Laboratories Inc., Burlingame, CA). Horse radish peroxidase was detected using the 3,3′-diaminobenzidine with metal enhancer reagent (Sigma Aldrich, St. Louis, MO). Images were acquired using an Olympus FSX100 at indicated magnifications.

### RNA isolation and quantitative real-time PCR

Total RNA from tissues of mice was isolated using the TriFast reagent according to the manufacturer’s protocol (Peqlab Biotechnologies GmbH, Erlangen, Germany). Two μg of total RNA was reverse transcribed using the High Capacity cDNA Reverse Transcription Kit (Applied Biosystems, Foster City, CA). Quantitative real-time PCR was performed on a Roche LightCycler 480 instrument (Roche Diagnostics, Palo Alto, CA) using the QuantiFast™ SYBR® Green PCR Kit (Qiagen, Valencia, CA). Samples were measured in triplicate for each experiment and normalized to *cyclophilin A* mRNA expression as an internal control. Expression profiles and associated statistical parameters were analyzed using the public domain program Relative Expression Software Tool – REST 2010 (http://www.gene-quantification.com/download.html) [19]. Primer sequences are available upon request.

### Western blotting

Livers from WTD-fed control and *GATA4*iKO mice were homogenized in radio-immunoprecipitation assay buffer and whole cell lysates were prepared and solubilized in Laemmli sample buffer. Proteins were resolved on precast Mini-PROTEAN–TGX gels (BioRad, Hercules, CA), blotted onto nitrocellulose membranes, blocked with 5% bovine serum albumin and probed with the appropriate polyclonal primary antibody overnight. After incubation with horseradish peroxidase-conjugated secondary antibody (Invitrogen, Carlsbad, CA), the immunoreactive proteins were detected using the enhanced chemiluminescence detection reagent (GE Healthcare, Piscataway, NJ). Antibodies against AMP-activated protein kinase (AMPK), phosphorylated (p)AMPK(Y^172^), acetyl-CoA carboxylase alpha (ACC), pACC(S^79^), p38 mitogen-activated protein kinase (p38), and p-p38(T^180^/Y^182^) were purchased from Cell Signaling Technology, Danvers, MA. Antibody against alpha smooth muscle actin (α-SMA) was purchased from Abcam, Cambridge, MA. For detection of the nuclear form of sterol regulatory element-binding protein 1 c (SREBP1c), 30 μg of hepatic nuclear extract prepared by using NE-PER nuclear cytoplasmic extraction reagents (Thermo Fisher Scientific, Pittsburgh, PA) was blotted and detected using an anti-SREBP1 antibody (Abcam, Cambridge, MA). The antibody against LaminA/C (Santa Cruz Biotechnology, Santa Cruz, CA) was a gift from Dr. W. Sattler (Medical University of Graz, Austria). Relative densities compared with controls were calculated from the percent area under the curve using Image J.

### Statistical analysis

Plasma parameters were compared using two parameter analysis of variance (Two-way ANOVA) followed by Bonferroni post-test. Hepatic TG, FFA, hepatic TBARS concentrations, and histochemical scorings were compared using the non-parametric Mann–Whitney test. Statistical significance for the oral fat tolerance was calculated using one way analysis of variance. Statistical analysis of mRNA expression levels REST 2010 was used [19]. Values are mean ± SE. ^∗^*p *<0.05, ^∗∗^*p *<0.01, ^∗∗∗^*p <*0.001.

## Results

### Reduced hepatic TG accumulation, SREBP-1c maturation, and lipogenic gene expression in *GATA4*iKO mice fed WTD

**Fig. 1:**
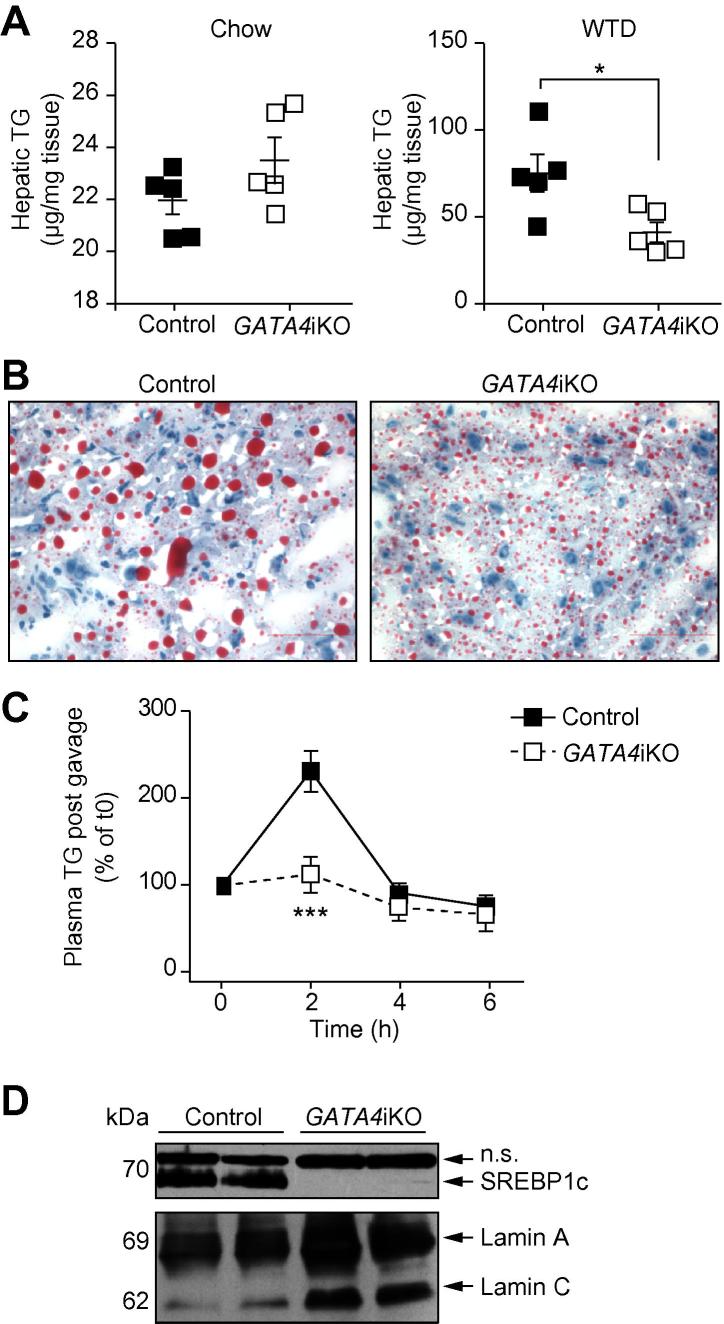
**Reduced hepatic triglyceride (TG) concentrations in Western-type diet (WTD)-fed *GATA4*iKO mice**. (A) Hepatic TG levels in Folch extracts of control and *GATA4*iKO mice fed chow and WTD (n = 5/group). (B) Representative Oil Red O stained images of liver sections from control and *GATA4*iKO mice fed WTD, showing TG (red) and nuclei (blue). (C) Plasma TG concentrations from three experiments (n = 5/group per experiment) of oral fat tolerance test expressed as percent of time 0. (D) Nuclear protein abundance of sterol response element-binding protein 1c (SREBP1c) and levels of lamin A/C as loading control. Values are means ± SE. ^∗^*p *<0.05, ^∗∗∗^*p *<0.001. n.s., not specific.

When fed a chow diet, *GATA4*iKO mice showed identical hepatic TG content (Fig. 1A). Twenty weeks of WTD feeding led to an increase in hepatic TG concentrations in both genotypes. *GATA4*iKO mice had a 44% reduction in hepatic TG levels compared with controls (Fig. 1A). These results were confirmed by Oil Red O staining of livers from WTD-fed control and *GATA4*iKO mice (Fig. 1B). Thus, the lack of intestinal GATA4 prevented diet-induced hepatic TG accumulation. To test for liver function, we measured serum concentrations of ALT, AST, and LDH and found them to be markedly reduced in *GATA4*iKO mice compared with controls (Table 1). WTD feeding also led to a decrease in body weight gain in *GATA4*iKO mice (Supplementary Fig. 1). To investigate the underlying mechanism of reduced hepatic TG accumulation, we first determined oral fat tolerance, which was prominently reduced in *GATA4*iKO mice (Fig. 1C). To compensate for the reduced intestinal uptake, we expected a proportional increase in hepatic DNL in *GATA4*iKO mice. Interestingly, hepatic *Srebp-1c* mRNA expression was reduced by 71% in WTD-fed *GATA4*iKO compared with control mice (Table 2). Livers from *GATA4*iKO animals lacked mature SREBP1c protein (Fig. 1D). Accordingly, hepatic mRNA expression of lipogenic targets of SREBP-1c was markedly decreased such as CD36 antigen, glycerol-3-phosphate acyltransferase 1, fatty acid synthase, acetyl-CoA carboxylase alpha, and stearoyl-CoA desaturase 1 in *GATA4*iKO compared with control mice (Table 2).

### Increased GLP-1 positive cells and AMPK activation in livers of *GATA4*iKO mice

**Fig. 2:**
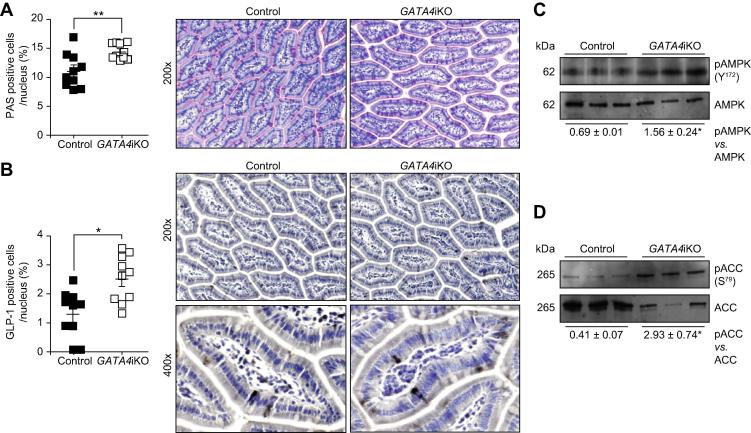
**Glucagon-like peptide 1 (GLP-1)-AMP-activated protein kinase (AMPK) pathway is active in *GATA4*iKO mice**. (A) Jejunal periodic acid Schiff (PAS) cells/nucleus expressed in percent. Representative images of sections stained with PAS to reveal secretory cell types (pink) and nuclei (blue) in control and *GATA4*iKO jejunum. (B) Jejunal GLP-1 positive cells/nucleus expressed in percent. Representative images of sections immunohistochemically stained for GLP-1 positive cells (black) and nuclei (blue) in control and *GATA4*iKO jejunum. (C) Protein expression of AMPK, phosphorylated pAMPK(Y^172^), (D) acetyl-CoA carboxylase alpha (ACC) and pACC(S^79^, inhibitory phosphate) in control and *GATA4*iKO livers. Numerical values below the blots represent mean relative densities. Values represent means ± SE. ^∗^*p <*0.05, ^∗∗^*p *<0.01.

Next, we investigated the mechanism by which the lack of intestinal GATA4 suppressed hepatic maturation of SREBP-1c and lipogenic gene expression. Previous reports in inducible intestine-specific *GATA4* knockout mice had shown that intestinal posteriorization led to a shift in cell type composition [15,20]. In agreement with these data, we observed a 1.4-fold increase in goblet cells in *GATA4*iKO jejuna [20] (Fig. 2A). To test whether intestinal posteriorization also led to an anterior increase in GLP-1 secreting L-cells, we stained for GLP-1, revealing 1.7-fold more GLP-1 positive cells in *GATA4*iKO compared with control jejuna (Fig. 2B). In accordance, jejunal transcript levels of *Glp-1* were 3.8-fold upregulated in *GATA4*iKO mice while hepatic expression of Glp-1 receptor was comparable in *GATA4*iKO and control livers (Table 2). Since GLP-1 is known to suppress hepatic lipogenesis via activation of AMPK [21], we determined hepatic phosphorylation of AMPK and its target ACC. As shown in Fig. 2C and D, hepatic phosphorylation of both AMPK(Y^172^) and ACC(S^79^) was increased in *GATA4*iKO compared with control mice. Consistent with our finding of suppressed *Acc* transcript levels (Table 2), we also found a dramatic reduction of ACC protein expression in *GATA4*iKO livers (Fig. 2D).

### Reduced hepatic FFA concentrations, lipid peroxidation, and fibrosis in WTD-fed *GATA4*iKO mice

**Fig. 3:**
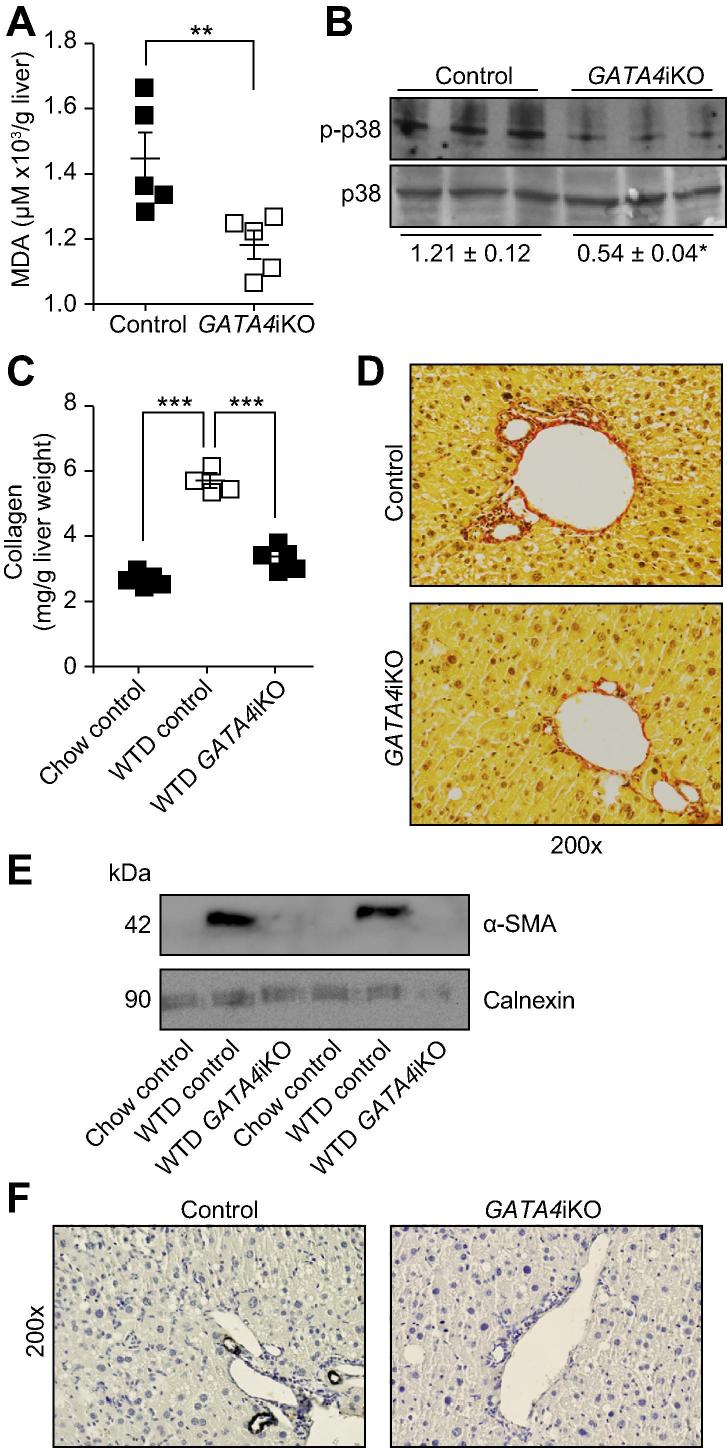
***GATA4*iKO mice have reduced hepatic lipid peroxidation and fibrosis upon 20 weeks of WTD feeding**. (A) Hepatic thiobarbituric acid reactive species levels determined as μM malondialdehyde adduct (MDA)/g liver tissue of control and *GATA4*iKO mice (n = 5/group). (B) Phosphorylated (T^180^/Y^182^) and total levels of the stress-induced p38 mitogen-activated protein kinase (p38) in livers from control and *GATA4*iKO mice. Numerical values below blots represent mean relative densities ± SE. (C) 4-Hydroxyproline concentrations converted into mg collagen/g liver (for conversion formula refer to methods) in chow-fed control, WTD-fed control and WTD-fed *GATA4*iKO mice (n = 4–5/group). (D) Representative images of liver sections stained for Sirius Red revealing collagen fibers (red), cytoplasm (yellow) and nuclei (gray). (E) Protein expression of alpha-smooth muscle actin (α-SMA) in livers from chow and WTD-fed control and WTD-fed *GATA4*iKO mice. Protein expression of calnexin was determined as loading control. (F) Representative images of liver sections immunostained for α-SMA (black) and nuclei (blue). Values are means ± SE. ^∗^*p <*0.05, ^∗∗^*p *<0.01, ^∗∗∗^*p <*0.001.

Hepatic accumulation of peroxidated lipids leads to oxidative damage contributing to hepatic injury [22]. We therefore determined the levels of FFA and TBARS in the liver. We found a 41% reduction in hepatic FFA concentrations (Supplementary Fig. 2) and a 19% decrease in hepatic lipid peroxidation (Fig. 3A) in WTD-fed *GATA4*iKO compared with control mice. As a result, *GATA4*iKO mice had lower levels of p38 phosphorylation (Fig. 3B).

To assess the fibrotic potential of WTD in our model, we determined 4-hydroxyproline concentrations (presented as mg collagen normalized to liver weight), Sirius Red staining and localization and protein expression levels of α-SMA. WTD led to a 2.2-fold increase in 4-hydroxyproline concentrations in control mice but levels were comparable in WTD-fed *GATA4*iKO and chow-fed controls (Fig. 3C). In agreement with these results, Sirius Red staining revealed reduced collagen fibers in *GATA4*iKO mice compared with controls (Fig. 3D). Protein expression of α-SMA was undetectable in liver homogenates of chow-fed controls, whereas WTD feeding caused a pronounced increase in α-SMA abundance in control but not *GATA4*iKO mice (Fig. 3E). Immunostaining for α-SMA was performed as a marker for myofibroblast and HSC activation. Staining was restricted around arterial walls and showed 16.3% positivity in WTD-fed *GATA4*iKO mice compared to 70.4% positivity in WTD-fed control mice (Fig. 3F middle and Supplementary Fig. 3). Thus, WTD led to a mild but significant hepatic fibrosis only in control mice. Hepatic transcript levels of collagen type I (73%), collagen type III (42%), tissue inhibitor of metalloproteinases 1 (69%), and actin*,* alpha-2 (53%) were downregulated in *GATA4*iKO compared with control mice (Table 2). The mRNA expression of peroxisome proliferator-activated receptor gamma (*Pparg*), a suppressor of HSC activation, was 7.1-fold upregulated in *GATA4*iKO mice (Table 2).

Haematoxylin and eosin staining indicated lower immune cell infiltration in WTD-fed *GATA4*iKO mice compared with controls (Supplementary Fig. 4). We also observed fewer CD45-positive cells in *GATA4*iKO mice (Supplementary Fig. 4). Transcript levels of the inflammatory cytokines tumor necrosis factor alpha (59%)*,* interferon gamma (57%), interleukin 1β (40%), interleukin 6 (48%), and interleukin 12 (42%) were also downregulated in WTD-fed *GATA4*iKO mice (Table 2).

### *GATA4*iKO fails to protect mice against MCDD-induced hepatic steatosis

**Fig. 4:**
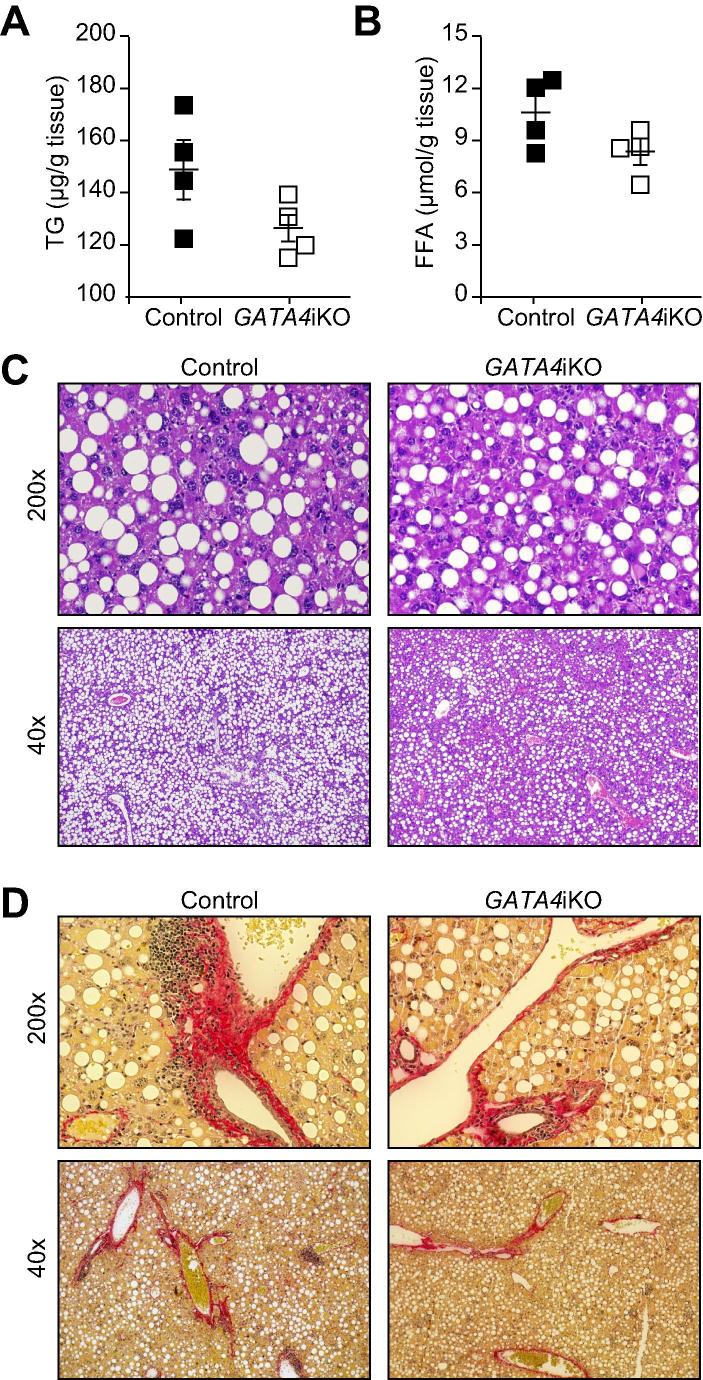
**Intestinal GATA4 deficiency does not protect from methionine and choline-deficient diet (MCDD)-induced hepatic steatosis**. Control and *GATA4*iKO mice (n = 4/group) were fed MCDD for 3 weeks. (A) Hepatic TG and (B) FFA concentrations determined in hepatic lipid extracts. (C) Representative images of liver sections stained for haematoxylin and eosin. (D) Representative images of liver sections stained for Sirius Red revealing collagen fibers (red), cytoplasm (yellow) and nuclei (gray). Values represent means ± SE.

We observed that the protective effects of *GATA4*iKO were absent when mice were challenged with MCDD. MCDD feeding led to a similar extent of weight loss (Supplementary Fig. 5) and hepatic injury in both genotypes as indicated by comparable levels of plasma AST, ALT, and LDH (Table 3). Hepatic accumulation of TG and FFA was unaffected (Fig. 4A and B). Assessment of liver damage as determined by haematoxylin and eosin as well as Sirius Red staining also indicated significant fat accumulation and fibrosis in both genotypes (Fig. 4C and D). In accordance, mRNA expression of genes involved in lipogenesis and fibrosis was unchanged (Table 4).

## Discussion

In this report, we show that WTD but not MCDD-fed *GATA4*iKO mice accumulate less hepatic TG and have reduced fibrosis than control mice. These findings suggest that intestinal deficiency of GATA4 has a role on hepatic metabolism and prevents diet-induced liver damage only in a setting that mimics metabolic disease. We had previously reported that *GATA4*iKO mice were resistant to diet-induced obesity on long-term WTD feeding [17]. A reduction in intestinal lipid absorption leads to a compensatory increase in hepatic DNL. Although *GATA4*iKO mice showed reduced intestinal TG absorption, hepatic DNL was suppressed. We speculate that GLP-1 leads to hepatic protection in *GATA4*iKO mice since TG-induced GLP-1 release is increased [17]. Hepatic protection by GLP-1 has also been demonstrated in genetic or dietary models that resemble obesity and metabolic syndrome but not under MCDD conditions. Therefore, GLP-1 release could be one of the endocrine mechanisms involved in the observed suppression of hepatic DNL in *GATA4*iKO mice. It has been reported that exendin-4, a GLP-1 receptor agonist, significantly reduces hepatic steatosis in genetically obese *ob/ob* mice and exerts a direct effect on the reduction of hepatic steatosis [23,24]. We found more GLP-1 positive cells and increased *GLP-1* mRNA expression in *GATA4*iKO jejuna. These findings are in good correlation with data obtained in rats that have undergone IIS [14]. GLP-1 is known to suppress hepatic lipogenesis via activation of the AMPK pathway [21]. In livers from *GATA4*iKO mice, increased intestinal GLP-1 secretion resulted in AMPK phosphorylation, leading to phosphorylation and inhibition of its target gene *ACC*, a key lipogenic enzyme. A recent study has shown that AMPK activation phosphorylates SREBP-1c at Ser372 and thus inhibits the cleavage and subsequent nuclear translocation of this lipogenic transcription factor [25]. Our results indicate AMPK-mediated inhibition of SREBP-1c maturation and subsequent downregulation of DNL being operative in livers of *GATA4*iKO mice. We conclude that apart from reduced intestinal absorption [17], GLP-1-mediated suppression of hepatic DNL is an important contributor to the observed hepatic protection in *GATA4*iKO mice.

The major cellular event in the development of liver fibrosis is the triggering of quiescent HSC into an active state [26]. Pparg is a known suppressor of HSC activation. Increased serum leptin levels have a net pro-fibrotic effect on the liver since leptin inhibits *Pparg* gene expression in HSC [27]. In contrast, adiponectin inhibits HSC proliferation and suppresses DNL by inhibiting SREBP1c maturation. Therefore, adiponectin attenuates progression of both alcoholic and NAFLD [28,29]. We also found decreased transcript levels of fibrotic and inflammatory marker genes and an increase of *Pparg* mRNA in the liver of *GATA4*iKO mice, indicating reduced HSC activation. *GATA4*iKO mice have decreased serum leptin (even on normal chow) and increased adiponectin concentrations [17,30]. We speculate that the leptin and adiponectin status in *GATA4*iKO mice leads to the observed upregulation in *Pparg* transcript levels in a HSC and not hepatocyte-specific manner. The effect of these adipokines on the activity and expression of hepatocyte Pparg is so far unknown. Expression of α-SMA, a marker of activated HSC, was reduced in *GATA4*iKO livers supporting our hypothesis that a favorable adipokine status in *GATA4*iKO mice may operate as an additional protective mechanism against fibrosis.

In summary, our results provide evidence that *GATA4*iKO in mice reduces intestinal TG uptake and lowers hepatic DNL through increased GLP-1 production. Our findings imply beneficial effects of *GATA4*iKO protecting against NAFLD and hepatic steatosis under conditions of metabolic syndrome.

## Financial support

This work was supported by the PhD Program “Molecular Medicine” of the Medical University of Graz, the Austrian Science Fund (P22832, SFB LIPOTOX F30, DK-MCD W1226 and P19186), the Austrian Federal Ministry of Science and Research (GEN-AU project Genomics of Lipid-associated Disorders-GOLD) and the Austrian National Bank (12929). Jay Patankar and Prakash Doddapattar were funded by and Sascha Obrowsky is a fellow of the PhD program “Molecular Medicine”.

## Conflict of interest

The authors who have taken part in this study declared that they do not have anything to disclose regarding funding or conflict of interest with respect to this manuscript.

## Figures and Tables

**Table 1:** **Plasma parameters of hepatic damage in WTD-fed *GATA4*iKO mice compared to controls**.

Values are means ± SE (n = 5/group). ^∗^*p* <0.05, ^∗∗^*p* <0.01.

**Table 2:** **mRNA fold change and *p* value for various genes in WTD-fed *GATA4*iKO mice compared to controls**.

Jej, Jejunum; Liv, Liver. Values are means ± SE (n = 5/group). ^∗^*p* <0.05 ^∗∗^*p* <0.01, ^∗∗∗^*p* *<*0.001.

**Table 3:** **Plasma parameters for hepatic damage in MCDD-fed *GATA4*iKO mice compared to controls**.

Values are means ± SE (n = 4/group).

**Table 4:** **mRNA fold change and *p* value for various genes in MCDD-fed *GATA4*iKO mice compared to controls**.

Values are means ± SE (n = 4/group).

## Appendix Supplementary data

Supplementary Figs. 1–5 This document file contains Supplementary Figs. 1–5.
