# Supplementary material for: Intestinal GATA4 deficiency protects from diet-induced hepatic steatosis
Source: J Hepatol. 2012 Nov;57(5):1061–8. doi: 10.1016/j.jhep.2012.06.028 (PMC3477492; doi:10.1016/j.jhep.2012.06.028)
Supplement: Supplementary Figs. 1–5 — This document file contains Supplementary Figs. 1–5. [file mmc1.doc]

**Supplementary Fig. 1. Reduced weight gain after western-type diet (WTD) feeding in GATA4iKO mice.** Percent body weight change in control and GATA4iKO mice fed WTD for 20 weeks compared to day 0. Values are means ± SE.*** *p <*0.001.

**Supplementary Fig. 2. GATA4iKO mice have reduced hepatic free fatty acid (FFA) concentrations.** FFA concentrations were determined in lipid extracts from WTD-fed control and GATA4iKO mice (n = 5/group) spectrophotometrically. Values are means ± SE.** *p <*0.01.

**A**


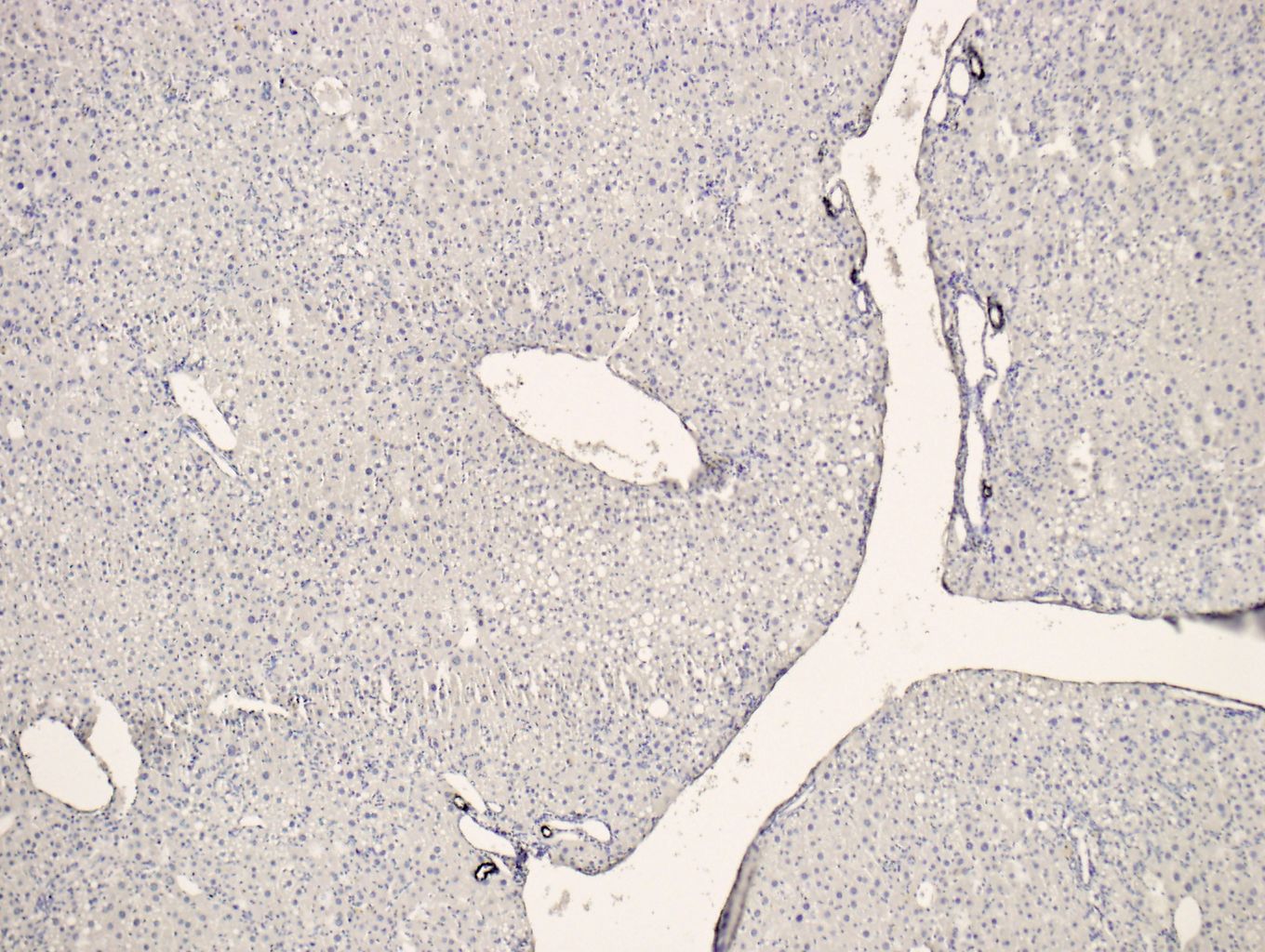

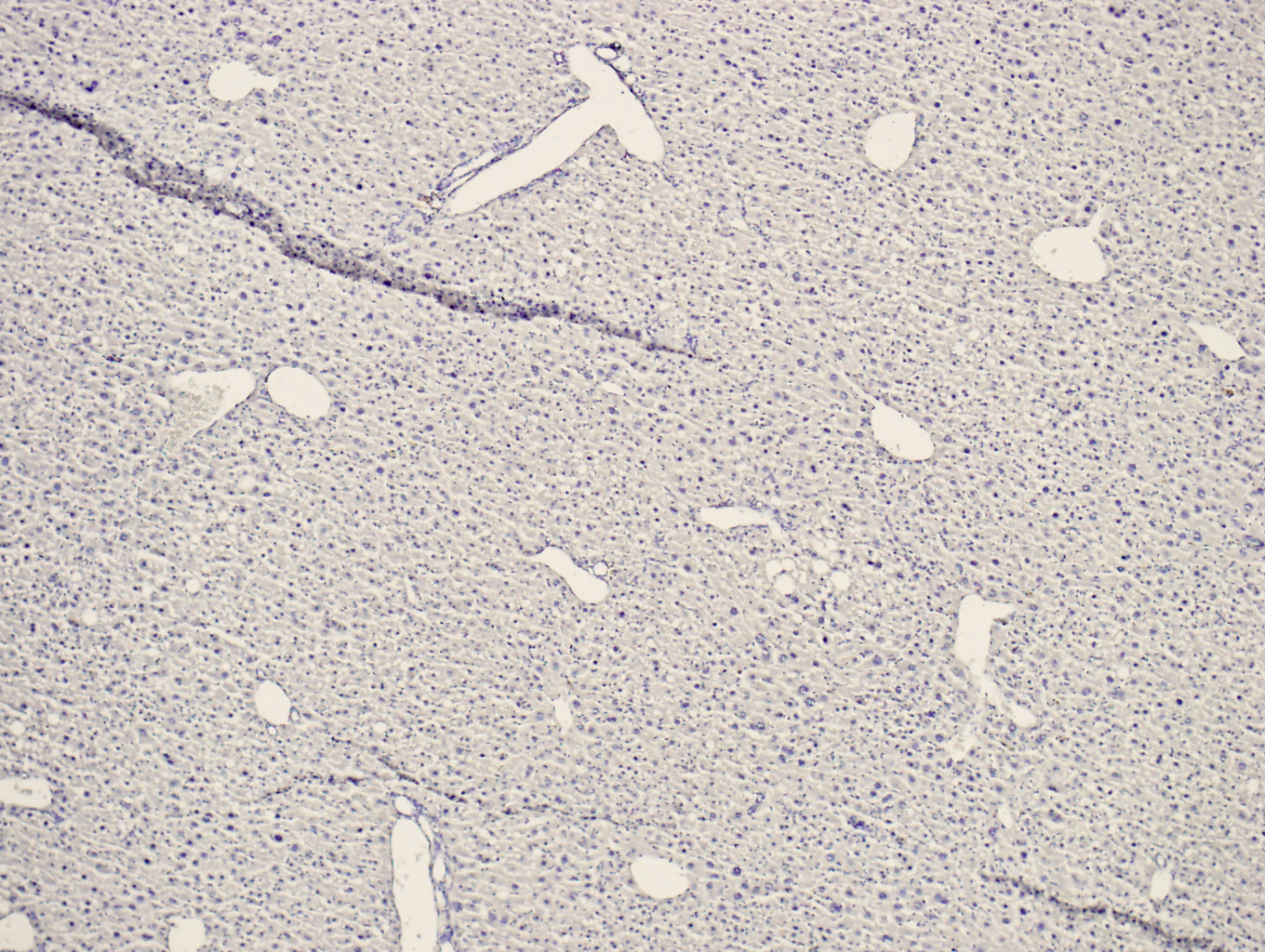
Control GATA4iKO

**B**

**Supplementary Fig. 3. Reduced α-SMA positivity in WTD-fed GATA4iKO mice.** Liver sections were immunostained for α-SMA and scored (n = 3/group). (A). Representative images show more positive cells around arteries in control compared with GATA4iKO knockouts. (B). Histogram shows percent positivity per genotype. Values are means ± SE.** *p <*0.01.

Control GATA4iKO

**
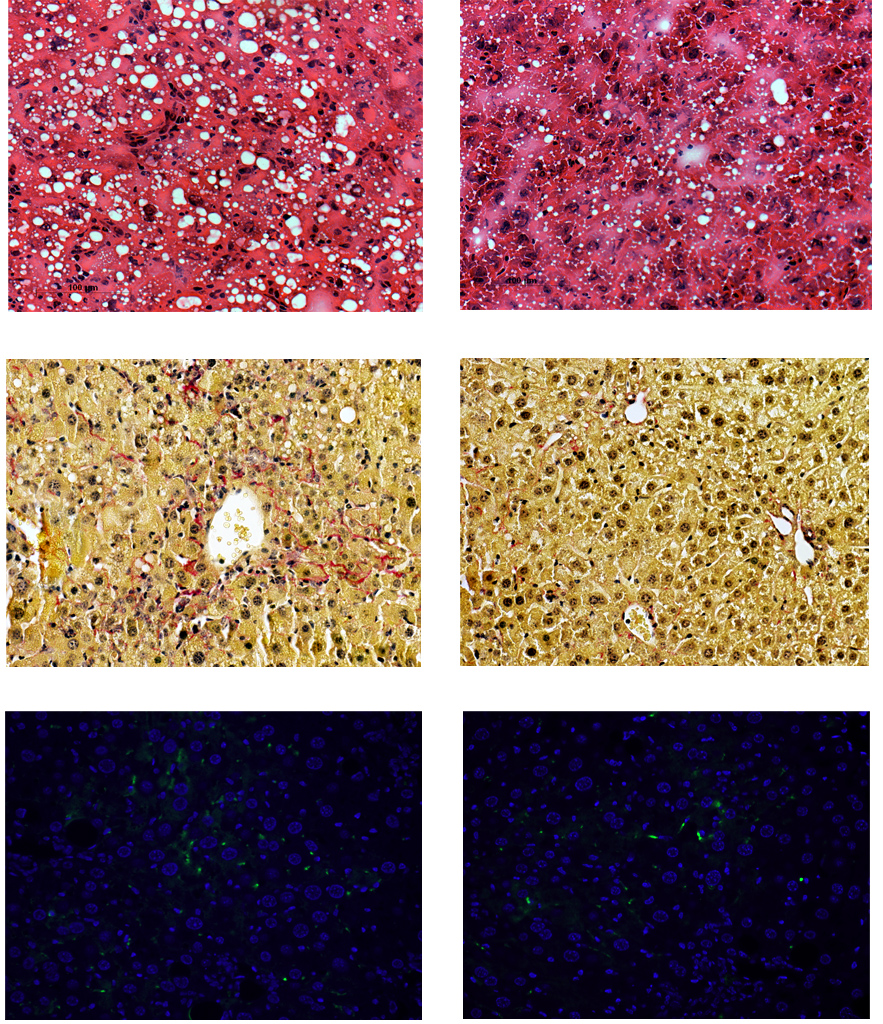
**

**
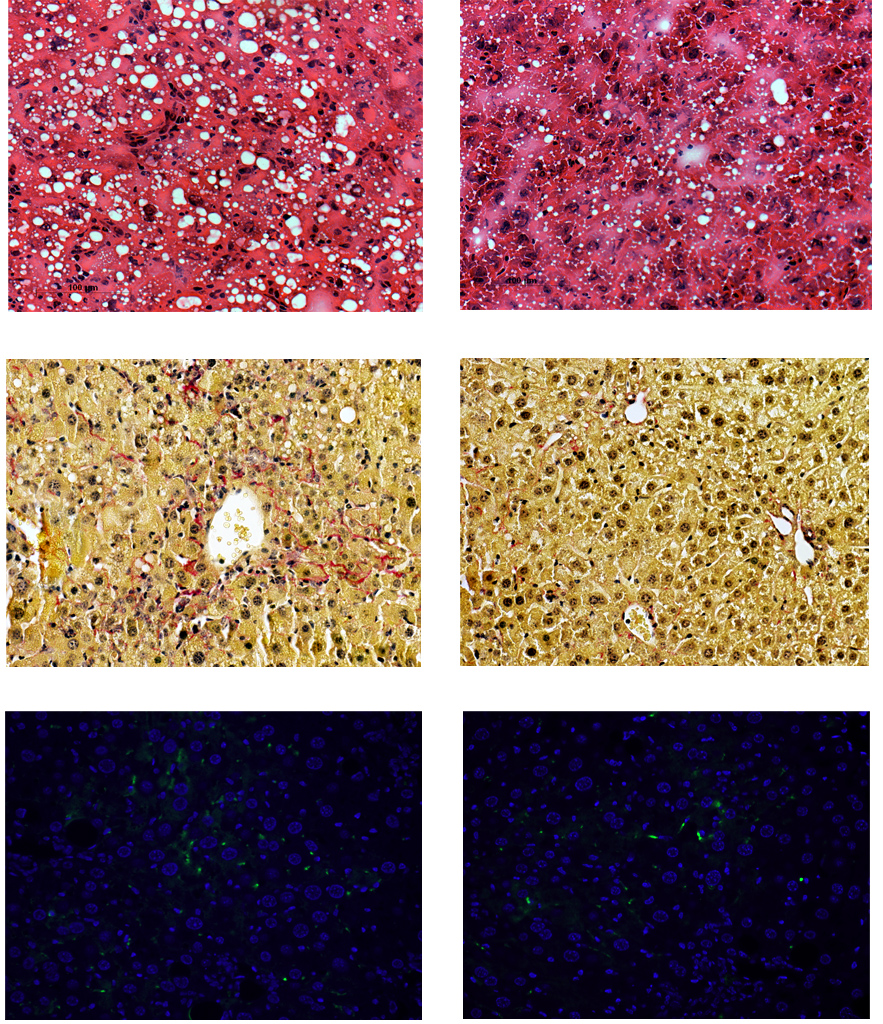
**

**Supplementary Fig. 4. Reduced steatosis and immune cell infiltration in WTD-fed GATA4iKO mice.** Representative images of liver sections were subjected to haematoxylin and eosin staining (*Top,* magnification: 200X).and CD45 immunostaining (*Bottom,* magnification: 200X).

**Supplementary Fig. 5. Comparable weight loss after methionine and choline-deficient diet (MCDD) feeding in GATA4iKO and control mice.** Percent body weight change in control and GATA4iKO mice fed MCDD for 3 weeks compared with day 0.
